# Cholecystocolic fistula closed using endoscopic therapy alone: A case report

**DOI:** 10.1097/MD.0000000000029680

**Published:** 2022-07-22

**Authors:** Kiyoyuki Kobayashi, Hideki Kobara, Tomohiro Ougi, Yuzuru Akaiwa, Takako Nomura, Maki Ougi, Kayo Ishikawa, Masafumi Ono, Hideki Kamada, Tsutomu Masaki

**Affiliations:** a Division of Innovative Medicine for Hepatobiliary and Pancreatology, Faculty of Medicine, Kagawa University, Kagawa, Japan; b Department of Internal Medicine, HITO Medical Center, Ehime, Japan; c Department of Gastroenterology and Neurology, Kagawa University, Kagawa, Japan.

**Keywords:** acute cholecystitis, case report, cholecystocolic fistula, endoscopic closure, endoscopic naso-gallbladder drainage

## Abstract

Cholecystocolic fistula (CCF) is a known but rare complication of cholelithiasis. Treatment for CCF is generally surgical. As the number of elderly patients has increased in recent years, many cases require non-surgical treatment; therefore, endoscopic treatment has gained importance.

**Patient concerns and diagnosis:**

An 87-year-old woman presented with impaired consciousness and symptoms of anorexia. Computed tomography showed cholecystitis and a fistula between the gallbladder and transverse colon. Colonoscopy revealed a CCF. The condition was diagnosed as CCF caused by acute cholecystitis.

**Interventions and outcomes:**

The patient declined surgery due to her age. Endoscopic fistula closure was performed using a through-the-scope clip after endoscopic naso-gallbladder drainage. Successful closure of the fistula resulted in improvement of cholecystitis and anorexia. The patient was discharged after one month. It has been more than 18 months since the procedure, there has been no recurrence.

**Conclusion:**

This report on successful endoscopic closure of a CCF indicates that it may be useful for patients who decline surgery.

## 1. Introduction

Internal biliary fistulas are rare, accounting for 0.4%–1.9% of all biliary tract diseases.^[1–3]^ Cholecystocolic fistula (CCF) is a rarer complication of cholelithiasis with cholecystitis, accounting for 8%–13.6% of internal biliary fistulas.^[1,4,5]^ Surgery is often performed to treat CCFs; however, it is associated with severe complications, such as acute cholangitis and biliary peritonitis.^[3,6,7]^ Owing to an aging population, there is an increasing number of elderly people who are unable to undergo surgical treatment due to the presence of various underlying diseases, or who refuse surgery based on their advanced age. Therefore, non-surgical treatment methods for CCFs may be in demand in the future. In this study, we reported a case of CCF where the patient was treated using endoscopic therapy alone.

## 2. Case report

An 87-year-old woman was transported to the hospital via an ambulance owing to impaired consciousness and anorexia for one week. The patient had a medical history of distal gastrectomy (Billroth I reconstruction), which was performed more than 10 years prior to this admission. The patient was a homemaker, had no history of smoking or drinking, and had no relevant family history. On admission, physical examination showed impaired consciousness with the Glasgow Coma Scale score of 13 and abdominal tenderness in the right hypochondrium. As head imaging identified no abnormalities, and the blood test results indicated dehydration, the patient was diagnosed with disorientation as a result of severe dehydration. We administered appropriate fluid infusion, and her disorientation had resolved the day after admission.

Complete laboratory data upon admission are shown in Table 1. Laboratory test results showed C-reactive protein level of 25.0 mg/L (normal: 0–0.14 mg/L), white blood cell count of 10000/μL (normal: 3300–8600/μL), hemoglobin level of 8.6 gm/dL (normal: 11.6–14.8 gm/dL), platelet count of 9.9 × 10^4^/µL (normal: 15.8–34.8 × 10^4^/µL), blood urea nitrogen level of 60.7 mg/dL (normal: 8.0–22.0 mg/dL), creatinine level of 1.44 mg/dL (normal: 0.41–0.76 mg/dL), carcinoembryonic antigen level of 5.3 ng/mL (normal: 0–5.0 ng/ml) and carbohydrate antigen 19–9 level of 8.6 U/mL (normal: 0–37.0 U/ml). Aminotransferase, alkaline phosphatase, gamma-glutamyl transpeptidase, and total bilirubin levels were within the normal ranges.

**Table 1:** Patient laboratory data upon admission.

Laboratory characteristics (parameters, measurement units)	Measurement values
WBC, /μL	10,000
N, %	95
Lymphs, %	4
M, %	1
EOS, %	0
Baso, %	0
RBC, 10^4^/μL	293
Hb, gm/dL	8.6
HCT, %	26.1
PLT, 10^4^/μL	9.9
TP, g/dL	5.1
Alb, g/dL	2.6
TBIL, mg/dL	0.7
AST, U/L	11
ALT, U/L	8
LDH, U/L	150
ALP, IU/L	65
GGT, U/L	21
AMS, U/L	113
BUN, mg/dL	60.7
Cr, mg/dL	1.44
Na, mEq/L	140
K, mmol/L	3.3
Cl, mEq/L	93
CRP, mg/L	25
CEA, ng/mL	5.3
CA19-9, U/mL	8.6

Alb = albumin, ALP = alkaline phosphatase, ALT = alanine aminotransferase, AMS = amylase, AST = aspartate aminotransferase, Baso = basophils, BUN = blood urea nitrogen, CA19-9 = carbohydrate antigen 19-9, CEA = carcinoembryonic antigen, Cl = chloride, Cr = creatinine, CRP = C-reactive protein, EOS = eosinophils, GGT = gamma-glutamyl transpeptidase, Hb = hemoglobin, HCT = hematocrit, K = potassium, LDH = lactate dehydrogenase, Lymphs = lymphocytes, M = monocytes, N = neutrophils, Na = sodium, PLT = platelets, RBC = red blood cell, TBIL = total bilirubin, TP = total protein, WBC = white blood cell.

**Figure 1.**
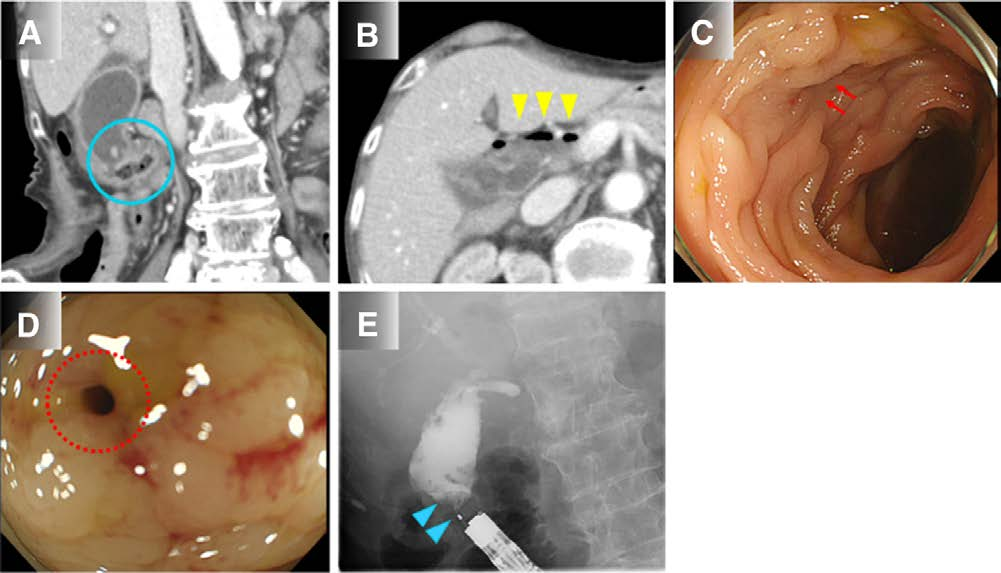
**Patient imaging before endoscopic closure.** (A) Abdominal contrast-enhanced computed tomography (CT). A suspected fistula is seen between the gallbladder and the transverse colon (blue circle). (B) Abdominal CT showed pneumobilia from the gallbladder to the common bile duct (yellow arrowhead). (C) Colonoscopy shows edematous changes in the mucosa of the transverse colon near the hepatic flexure, and a fistula could be seen between the folds (red arrow). (D) The fistula with a diameter of 3–4 mm was confirmed (red dotted circle). (E) When contrast medium was injected into the fistula, the gallbladder and bile duct were easily visualized (blue arrowhead).

Swelling of the gallbladder, thickening of the gallbladder wall, peri-gallbladder abscess, gallstones, and pneumobilia were visible on abdominal contrast-enhanced computed tomography (CT) images, in addition to a suspected fistula between the gallbladder and transverse colon (Fig. 1A,B). A fistula, 3–4 mm in diameter, in the transverse colon near the hepatic flexure, was found during colonoscopy (Fig. 1C,D). The gallbladder and bile duct were easily visualized when a contrast medium was injected into the fistula using an endoscopic retrograde cholangiopancreatography (ERCP) catheter (ERCP-Catheter; MTW) (Fig. 1E). The endoscopic findings were not suggestive of a malignant tumor; however, a fistula biopsy was collected by pressing against the inside of the fistula using forceps (Radial Jaw™ 4 Jumbo Cold Polypectomy Forceps; Boston Scientific) and bile juice cytology was performed with the bile juice collected from the ERCP catheter to rule out fistula formation due to the malignant tumor. No tumor cells were found in the biopsy or cytology results.

Therefore, based on laboratory, imaging, and pathology findings, the patient was diagnosed with a CCF located between the gallbladder and the transverse colon, caused by acute cholecystitis. *Enterococcus faecalis* was detected in the bile culture, and the infection was treated with tazobactam and piperacillin hydrate.

**Figure 2.**
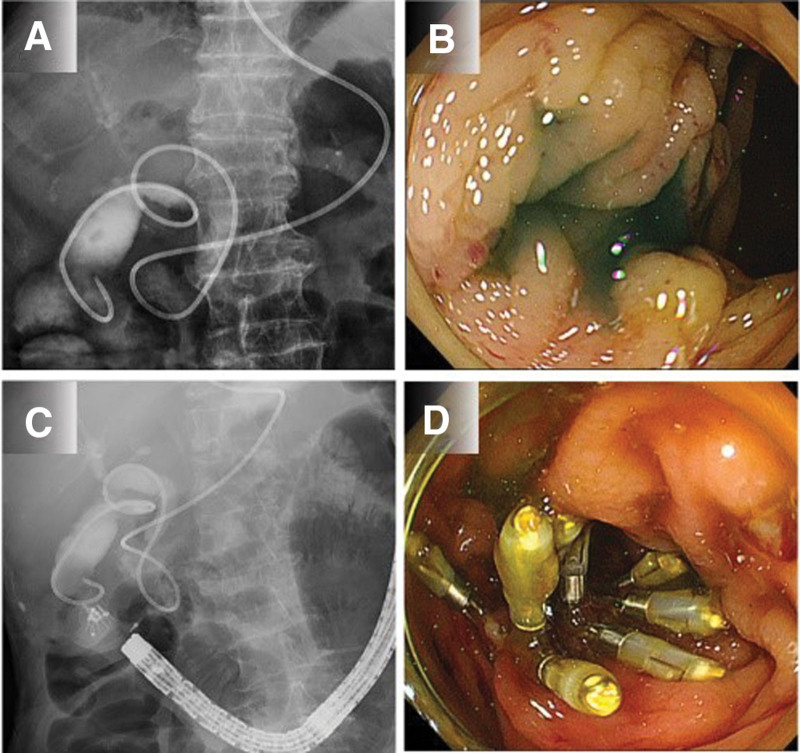
**Indigo carmine injection images and endoscopic closure.** (A) Endoscopic naso-gallbladder drainage (ENGBD) was performed, and bile was drained from the gallbladder. (B) Injection of indigo carmine through the ENGBD tube was visualized draining from the fistula into the transverse colon. (C) Endoscopic fistula closure was performed with through-the-scope clips. (D) After endoscopic closure, the outflow of contrast medium and indigo carmine ceased.

Although the surgical department recommended surgical treatment based on the examination results, the patient and her family refused surgery due to her advanced age and concerns regarding her ability to endure the procedure. Based on the patient’s wishes, we performed endoscopic naso-gallbladder drainage (ENGBD) to treat the acute cholecystitis and close the fistula. Fistula closure was performed as the colonoscopy showed that fecal matter was flowing into the gallbladder via the fistula. We thought that ENGBD alone would not stop the inflow of fecal matter into the biliary tract and would inhibit the closure of the fistula, so we considered that endoscopic closure of the fistula was necessary in addition to ENGBD. After receiving informed consent from the patient and the family, ENGBD was performed to treat acute cholecystitis and prevent bile exposure to the fistula (Fig. 2A). Endoscopic fistula closure was performed after ENGBD. When the endoscope (PCF-Q260AZI; Olympus) was positioned near the fistula, the outflow of indigo carmine, infused through the ENGBD tube, from the site of the fistula was confirmed (Fig. 2B). Endoscopic fistula closure was performed using six through-the-scope clips ([TTSCs] EZ Clip, HX-610-090L; Olympus). The fistula was closed with the clips, and it was confirmed that the fistula could no longer be visualized with imaging when a contrast medium was injected through the ENGBD tube (Fig. 2C,D). The patient was started on a liquid diet.

**Figure 3.**
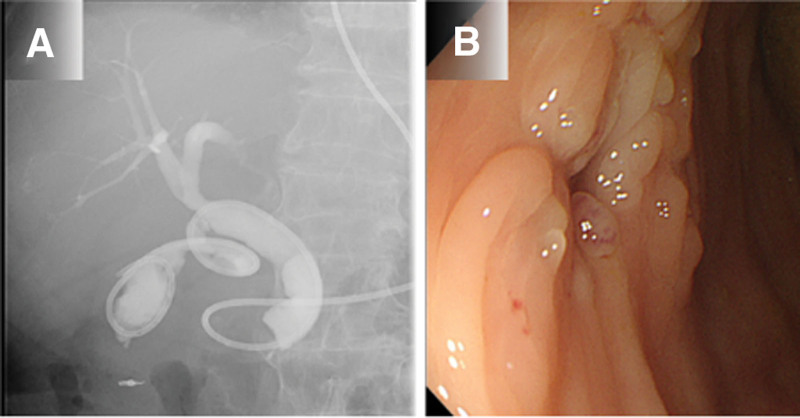
**Patient imaging after endoscopic closure.** (A) Fourteen days after closure, there was no visible outflow of contrast medium from the endoscopic naso-gallbladder drainage (ENGBD) tube into the transverse colon. (B) Fourteen days after closure confirmation, the fistula closure was confirmed endoscopically.

**Figure 4.**
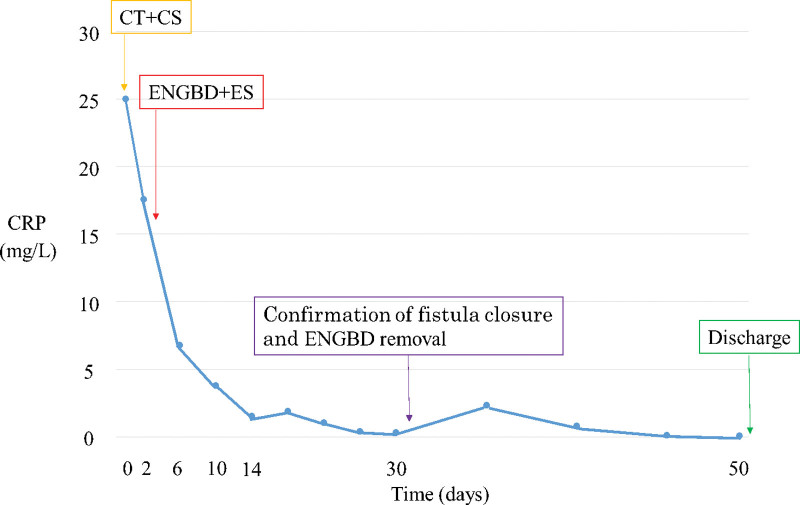
**The timing of each endoscopic procedure and changes in the levels of C-reactive protein during the clinical course.** CT = abdominal contrast-enhanced computed tomography, CS = colonoscopy, ENGBD = endoscopic naso-gallbladder drainage, ES: endoscopic closure.

Fourteen days post endoscopic fistula closure, the fistula and colon could not be visualized with imaging after the injection of contrast medium through the ENGBD tube (Fig. 3A). A further 14 days later, the fistula was no longer visible during colonoscopy and there was no outflow of indigo carmine when this was injected through ENGBD tube; thus, fistula closure was confirmed (Fig. 3B). Subsequently, the patient’s symptoms improved, and she could consume a normal diet. The ENGBD tube was removed 28 days post-procedure, and the patient was discharged on day 52 (Fig. 4). It has been more than 18 months since the procedure. There has been no recurrence. We plan to continue follow-up with close monitoring for the recurrence of acute cholecystitis.

## 3. Discussion

We reported a case of successful CCF closure with endoscopic treatment alone. Based on the literature and to the best of our knowledge, this is the first such case reported.

Cholecystocolic fistula is rare and can be caused by a variety of diseases. Cholecystolithiasis causes adhesion to the gallbladder and surrounding organs due to obstruction and recurrent infections, resulting in CCFs, leading to additional inflammation, which is reported at a rate of 0.1% in patients with the biliary disease.^[8–10]^ Additionally, CCFs can be caused by abdominal trauma, Crohn’s disease, and malignancies of the biliary tract, colon, and head of the pancreas.^[11]^ Therefore, some cases have been misdiagnosed as colon cancers in the past.^[11,12]^ Plain abdominal radiography, barium enema, ultrasound, colonoscopy, abdominopelvic CT scan, and/or diagnostic laparotomy may be used to diagnose CCFs.^[13,14]^ Moreover, CCF should be suspected when pneumobilia is observed in patients with no history of endoscopic sphincterotomy, such as in this case, even if there is no visible fistula on CT images. Nevertheless, diagnosis by imaging alone is challenging, and less than 10% of cases can be diagnosed preoperatively.^[9,10]^ The symptoms of CCFs are often non-specific and include diarrhea, melena, and weight loss. In this case, there were some non-specific symptoms, such as impaired consciousness and abdominal tenderness in the right hypochondrium, but no specific symptoms of CCF were observed. However, a CCF was strongly suspected on the CT scan, and the presence of a fistula was confirmed by colonoscopy. The presence of gallbladder stones and a peri-gallbladder abscess suggested that the cause of the present patient’s CCF was acute cholecystitis rather than a malignant disorder. However, the possibility of malignant disorders could not be ruled out, so a biopsy was taken from the fistula area and bile cytology was performed. The results of these investigations identified no malignant cells. The patient did not wish to undergo surgery and the absence of malignancy was confirmed by pathological examination, so endoscopic treatment was chosen rather than gallbladder resection.

Open cholecystectomy and fistula closure are standard treatments for CCF; however, in recent years, treatment with laparoscopic surgery has also been reported with good results.^[8,9,15]^ In contrast, endoscopic fistula closure has been reported as a treatment for gastrointestinal (GI) fistulas, although it has never been reported for CCFs. Clips are the most commonly used endoscopic devices to treat GI perforations and fistulas.^[16]^ There are two types of endoscopic clips: TTSCs and over-the-scope clips (OTSCs). TTSCs are technically easy and ready to use during endoscopy; however, they can be used only for small defects (<1 cm). In contrast, OTSCs provide full-thickness closure of defects up to 2 cm with a single application.^[17]^ Although OTSCs can be closed with suction for a small fistula with little fibrosis, the technical issues of OTSCs include the need to completely withdraw the endoscope to mount the device to the tip and that they sometimes require the use of assistive devices for more accurate clipping of large areas.^[18]^ Additionally, modifications after OTSC use may not be possible because OTSC removal can be challenging.^[19,20]^ In this case, the patient and the family refused surgery; therefore, an endoscopy was performed. We performed ENGBD for biliary drainage rather than percutaneous transhepatic gallbladder drainage because the gallbladder was not full owing to the presence of the CCF. Through-the-scope clips were used in this case because the fistula was small (3-4 mm in diameter). Through-the-scope clips are readily available, inexpensive, and their positioning can be corrected after clip placement. The average clinical success rate of fistula closure using OTSCs is as low as 52%; however, there are reports that the clinical success rate is more likely to be achieved for fistulas with a diameter of less than 10 mm.^[21]^ Therefore, if fistula closure was not possible with TTSCs, we had planned to use an OTSC to complete the procedure. As the fistula was successfully closed with endoscopic treatment alone; the patient subsequently resumed normal food intake and was ultimately discharged. The present case showed that endoscopic suturing could be an alternative treatment for inoperable cases of CCF.

In conclusion, CCFs are a rare complication of cholecystitis, although they may be caused by malignant tumors. The number of elderly patients will continue to increase in the future, and the number of inoperable cases of CCF is also expected to increase. Although surgery is the first treatment choice, we suggest that endoscopic biliary drainage and fistula closure are suitable treatment options for inoperable cases of CCF.

## Acknowledgments

We would like to thank Editage (www.editage.com) for English language editing.

## Author contributions

KK and KH designed the report. KK, OT, AY, NT, OM, and IK were in charge of the treatment of the patient and contributed to the collection of clinical data. OM was responsible for the analysis and interpretation of the data. KK analyzed the data and wrote the manuscript. KH and KH reviewed and edited the manuscript. MT was responsible for the study supervision. All authors were involved in data interpretation and drafting the manuscript and have read and approved the final version of the manuscript.
